# Supplementary material for: Research Trends and Hotspots of Retinal Optical Coherence Tomography: A 31-Year Bibliometric Analysis
Source: J Clin Med. 2022 Sep 23;11(19):5604. doi: 10.3390/jcm11195604 (PMC9572389; doi:10.3390/jcm11195604)
Supplement: Supplementary file 1 [file jcm-11-05604-s001.zip › jcm-1898977-supplementary.pdf]

Table S1. Cluster 1 of the top 100 keywords.

Rank	Keywords	Counts
1	optical coherence tomography	11305
2	eyes	2716
3	thickness	2313
4	glaucoma	1958
6	nerve-fiber layer	1765
8	OCT	1663
13	prevalence	1167
19	choroidal thickness	1005
20	reproducibility	1005
24	progression	908
27	open-angle glaucoma	818
35	damage	712
36	coherence tomography	689
37	retinal nerve fiber layer	672
38	age	653
41	retinal thickness	620
44	association	575
45	risk-factors	575
47	diagnosis	567
48	intraocular-pressure	555
50	axial length	534
57	myopia	483
63	fiber layer thickness	470
65	macular thickness	465
66	children	461
70	atrophy	437
85	multiple-sclerosis	396
86	abnormalities	390
87	thickness measurements	371
94	head	358
98	multiple sclerosis	352
99	population	351

Table S2. Cluster 2 of the top 100 keywords.

Rank	Keywords	Counts
7	macular degeneration	1691
9	retinopathy	1530
10	ranibizumab	1446
12	choroidal neovascularization	1172
16	age-related macular degeneration	1039
17	macular edema	1034
18	visual-acuity	1020
22	endothelial growth-factor	933
23	bevacizumab	924
26	photodynamic therapy	822
29	therapy	784
32	diabetic macular edema	742
33	central serous chorioretinopathy	732
52	edema	519
55	injection	508
58	indocyanine green angiography	482
64	neovascularization	467
67	management	456
69	verteporfin	439
75	secondary	424
80	uveitis	404
84	intravitreal bevacizumab	398
88	efficacy	369
91	cystoid macular edema	360
92	expression	360

Table S3. Cluster 3 of the top 100 keywords.

Rank	Keywords	Counts
15	degeneration	1055
21	disease	948
34	fundus autofluorescence	731
42	in-vivo	603
46	eye	572
53	segmentation	513
56	classification	491
60	geographic atrophy	479
68	images	451
74	maculopathy	426
78	mutations	407
81	high-speed	403
82	retinal-pigment epithelium	403
83	retinitis-pigmentosa	400
90	spectral-domain optical coherence tomography	361
93	sensitivity	359
95	microperimetry	358
96	model	357
100	resolution	346

Table S4. Cluster 4 of the top 100 keywords.

Rank	Keywords	Counts
5	retina	1904
11	optical coherence tomography angiography	1175
25	fluorescein angiography	848
30	angiography	776
31	diabetic retinopathy	753
39	blood-flow	652
40	features	628
51	diabetic-retinopathy	534
54	macula	509
59	choroid	480
61	coherence tomography angiography	476
62	foveal avascular zone	474
72	risk	430
73	vessel density	428
76	imaging	422
79	oct angiography	405
89	choriocapillaris	361

Table S5. Cluster 5 of the top 100 keywords.

Rank	Keywords	Counts
14	vitrectomy	1134
28	surgery	784
43	outcomes	591
49	macular hole	537
71	epiretinal membrane	430
77	internal limiting membrane	417
97	pathogenesis	357

Table S6. Details of clusters.

Cluster	Size	Silhouette	Mean (Year)	Label (log-likelihood ratio algorithm)
0	297	0.889	2015	optical coherence tomography angiography
1	193	0.93	2005	ophthalmology
2	190	0.954	1994	partial coherence interferometry
3	183	0.888	2006	bevacizumab
4	173	0.932	2011	glaucoma
5	171	0.942	2002	scanning laser polarimetry
6	135	0.971	2011	choroidal thickness
7	130	0.866	2003	retinal thickness
8	120	0.955	1998	macular hole
9	115	0.956	2015	deep learning
10	114	0.981	2011	multiple sclerosis
11	102	0.897	2009	epiretinal membrane
12	101	0.943	2012	reticular pseudodrusen
13	94	0.956	2017	choriocapillaris
14	93	0.979	2015	Alzheimer's disease
15	84	0.957	2015	central serous chorioretinopathy
16	75	0.976	2016	vessel density
17	63	0.981	2012	adaptive optics
18	53	0.95	2008	retinitis pigmentosa
19	53	0.976	2003	triamcinolone acetonide
20	52	0.952	2012	aflibercept
21	50	0.976	2001	photodynamic therapy
22	45	0.995	2012	vitreomacular traction
23	44	0.995	2014	diabetic macular edema
25	9	1	2004	electroretinogram
27	7	1	1992	resolution

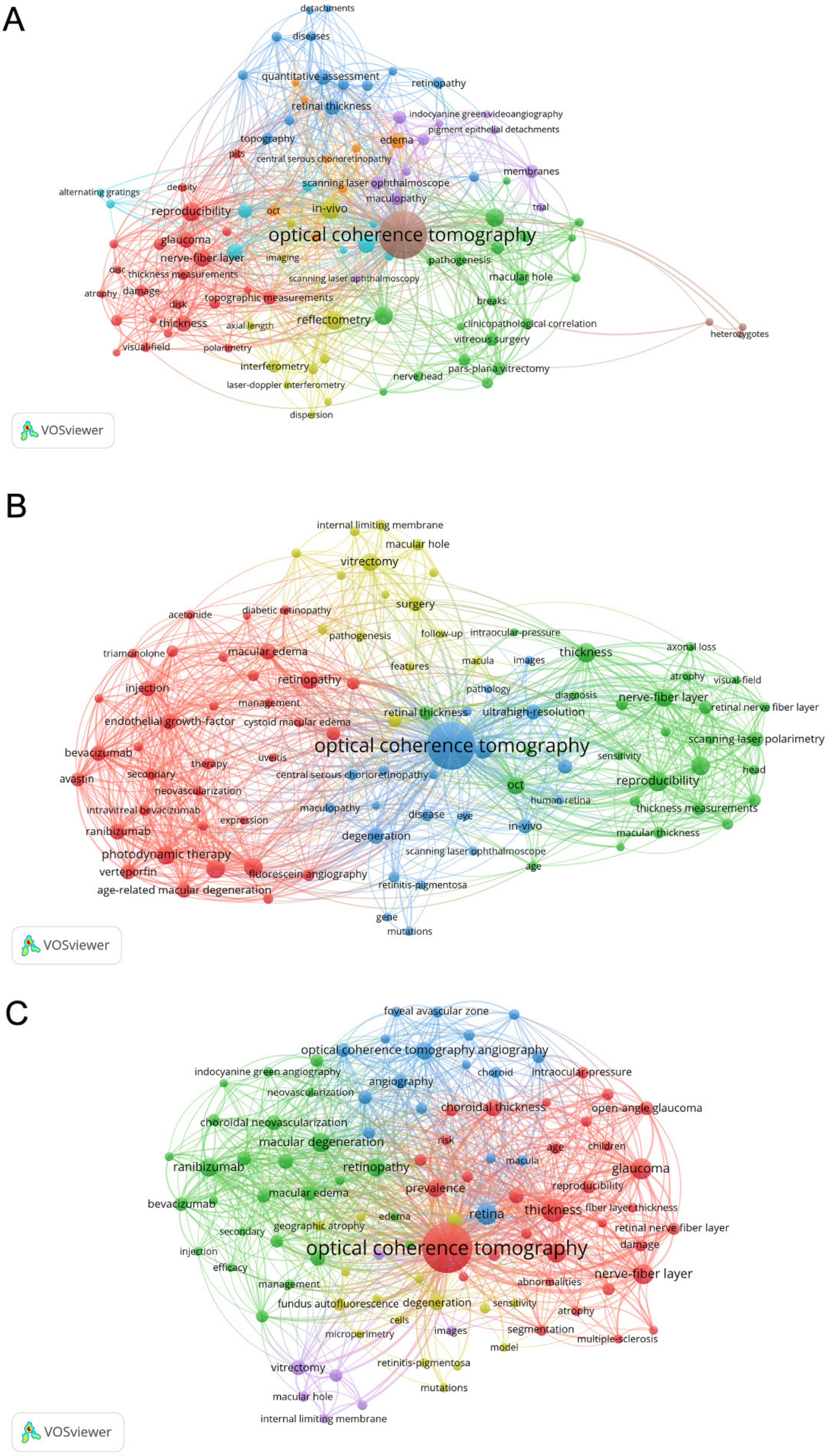

Figure S1. Co-occurrence analysis of the top 100 keywords. (**A**) Co-occurrence network for publications in the first decade (1991-2001); (**B**) Co-occurrence network for publications in the second decade (2002-2011); (**C**) Co-occurrence network for publications in the third decade (2012-2021).
